# QKI deficiency leads to osteoporosis by promoting RANKL-induced osteoclastogenesis and disrupting bone metabolism

**DOI:** 10.1038/s41419-020-2548-3

**Published:** 2020-05-07

**Authors:** Tianshu Du, Zhao Yan, Shu Zhu, Guo Chen, Li Wang, Zichen Ye, Wenwen Wang, Qingsheng Zhu, Zifan Lu, Xiaorui Cao

**Affiliations:** 1 PLA Institute of Orthopaedics, Xijing Hospital, Fourth Military Medical University, No.17, Changle West Road, Xincheng District, Xi’an, Shaanxi Province China; 2 State Key Laboratory of Cancer Biology, Department of Pharmacogenomics, Fourth Military Medical University, No.17, Changle West Road, Xincheng District, Xi’an, Shaanxi Province China

**Keywords:** Mechanisms of disease, Transcriptional regulatory elements

## Abstract

Quaking (QKI), an RNA-binding protein, has been reported to exhibit numerous biological functions, such as mRNA regulation, cancer suppression, and anti-inflammation. However, little known about the effects of QKI on bone metabolism. In this study, we used a monocyte/macrophage-specific QKI knockout transgenic mouse model to investigate the effects of QKI deficiency on receptor activator of NF-κB ligand (RANKL)-induced osteoclastogenesis. The loss of QKI promoted the formation of multinucleated tartrate-resistant acid phosphatase (TRAP)-positive osteoclasts (OCs) from bone marrow macrophages, and upregulated the expression of OC-specific markers, including TRAP (Acp5) and cathepsin K (Ctsk). The pro-osteoclastogenesis effect of QKI deficiency was achieved by amplifying the signaling cascades of the NF-κB and mitogen-activated protein kinase (MAPK) pathways; then, signaling upregulated the activation of nuclear factor of activated T cells c1 (NFATc1), which is considered to be the core transcription factor that regulates OC differentiation. In addition, QKI deficiency could inhibit osteoblast (OB) formation through the inflammatory microenvironment. Taken together, our data suggest that QKI deficiency promoted OC differentiation and disrupted bone metabolic balance, and eventually led to osteopenia under physiological conditions and aggravated the degree of osteoporosis under pathological conditions.

## Introduction

Bone is a rigid connective tissue that possesses important functions, such as protecting various organs, storing minerals, and harboring bone marrow^1^. Bone is also a highly dynamic organ because of its continuous remodeling. Although the bone-forming OB synthesizes and mineralizes the bone extracellular matrix (ECM), the bone-resorbing OC is responsible for resorbing this mineralized ECM^2^. The maintenance of bone homeostasis is dependent on the balance of the activities of OB and OC. Any abnormal bone remodeling process causes various skeletal disorders, such as osteoporosis, osteonecrosis, and osteolysis^3^. These diseases would deteriorate the bone microarchitecture, decrease the bone mass, and ultimately increase fracture risk^4^. As the only cell type well accepted to resorb bone in the human body, OCs have a key role in skeletal health.

OCs are multinucleated giant cells that originate from mononuclear myeloid hematopoietic stem cells of bone marrow and are formed by the fusion of multiple monocytes/macrophages^5^. Macrophage colony-stimulating factor (M-CSF) activation of its receptor c-Fms and RANKL activation of its receptor RANK are important signaling events that prompt OC precursors proliferation and differentiation^4^. RANKL signaling activates transcription factors, such as NF-κB, NFATc1, c-Fos, and calcineurin (CN), through triggering various downstream MAPK signaling cascades, such as p38, c-Jun N-terminal kinase (JNK), and extracellular signal-regulated kinase (ERK) pathways, to upregulate OC functional genes, such as TRAP and Ctsk, which are considered the readouts of OC bone resorption and the marker genes of OCs^2,5–9^. However, our understanding of the signaling pathways that govern OC differentiation is far from complete.

Quaking (QKI) is a member of the signal transduction and activator of RNA metabolism (STAR) and hnRNP K homology-type family of RNA-binding proteins^10^. Substantial research implicated QKI RNA-binding protein function in many more cell types than initially anticipated. Like many mRNA regulators, quaking-related proteins regulate the expression of diverse mRNA targets by various mechanisms and have essential roles in cell cycle and differentiation regulation^11–20^. Some reports have recently indicated that QKI significantly influences macrophage differentiation and polarization^21–23^. We have previously shown a novel role for QKI in restraining immune responses in mice by favoring the anti-inflammatory (M2) polarized macrophages rather than the pro-inflammatory (M1) polarized macrophages and revealed that QKI was a potent inhibitor of the NF-κB pathway, suppressing the latter isoform p65 expression and phosphorylation^23^.

Given the prominent activities of QKI in the monocyte/macrophage lineage and the original role of monocyte/macrophage lineage in osteoclastogenesis, we speculated a potential function of QKI in osteoclastogenesis. In our present study, we demonstrated that QKI has a critical role in the regulation of osteoclastogenesis in mice with a normal physiology and bone-associated pathology. Using genetic mouse models in vitro and vivo, we uncovered that a specific deficiency of QKI in the myeloid lineage promoted OC differentiation by activating the RANKL-induced NF-κB and MAPK pathways.

## Materials and methods

### Mouse model

Generation of KO mice was reported previously^23^. All mouse experiments and procedures were approved by the Laboratory Animal Center of Air Force Military Medical University and conducted in compliance with the ethical standards.

### Materials

Alpha-modification of Eagle medium (α-MEM) and penicillin/streptomycin were purchased from HyClone (Logan, UT, USA), and fetal bovine serum (FBS) was purchased from Gibco (USA). Recombinant Murine M-CSF (#315-02) was purchased from PeproTech (Rocky Hill, USA), and recombinant mouse RANKL (#462-TEC-010) was purchased from R&D Systems (USA). Rabbit polyclonal antibody against QKI was purchased from Sigma Aldrich (St Louis, MO, USA). Rabbit polyclonal antibodies against OCN (GB11233), RANKL (GB11235), OPG (GB11151), IL-1β (GB11113), TNF-α (GB11188), and a biotinylated goat anti-rabbit secondary antibody (G23303) were purchased from Servicebio (Wuhan, China). Mouse monoclonal antibodies against NFATc1 (7A6): sc-7294, c-Fos (6-2H-2F): sc-447 and TRAF6 (D-10): sc-8409 were purchased from Santa Cruz Biotechnology (USA). Rabbit monoclonal antibodies against NF-κB p65 (#8242), phospho-NF-κB p65 (Ser536) (#3033), p38 (#8690), phospho-p38 (Thr180/Tyr182) (#9211), c-JNK (#9252), phospho-JNK (Thr183/Tyr185) (#9251), ERK (#4695), phospho-ERK (Thr202/Tyr204) (#9101), and β-actin (#8457) were purchased from Cell Signaling Technology (Cambridge, MA, USA). Rabbit polyclonal antibodies against CN (WL00981), IκBα (wl01936), and phospho-IκBα (Ser32/Ser36) (WL02495) were purchased from Wanleibio (Shenyang, China). BAY11-7082, SB203580, FR180204, and SP600125 were purchased from Tsbiochem (Shanghai, China). The TRAP staining kit and Alizarin red staining kit were purchased from Sigma Aldrich (St Louis, MO, USA). The mouse Acp-5b ELISA kit and mouse OCN ELISA kit were purchased from Cloud-Clone (Wuhan, China). A TRAP activity microplate test kit was purchased from JianCheng Bioengineering (NanJing, China).

### Methods

#### µCT imaging analysis

The fixed femurs from KO and control mice were analyzed using a high-resolution µCT scanner (GE-LSP, USA). The experimental mice included four male and two female KO mice, four male and two female control mice at 8 weeks old, three male and two female KO mice, three male and two female control mice at 36 weeks old. All femurs were scanned according to the same parameters (tube voltage, 80 kV; tube current, 80μA; exposure time, 3000 ms; effective pixel size, 8 μm). The further structural and quantitative parameters were collected by GE Healthcare software following the nomenclature describes by the American Society for Bone and Mineral Research Nomenclature Committee. Trabecular morphometry was determined by measuring the BMD, BV/TV, BVF, Tb.Th, Tb.N, Tb.Sp, and SMI.

#### Immunohistochemistry (IHC) and histological analysis

After µCT scanning, the samples were decalcified in 10% EDTA for 20 days and then dehydrated and embedded in paraffin. Histological sections (5 μm thick) were prepared for H&E and TRAP staining. The rest were prepared for IHC. Briefly, the slides were blocked with 3% BSA and were incubated with anti-QKI (1:500), anti-OCN (1:100), anti-RANKL (1:100), anti-OPG (1:200), anti-IL-1β (1:500), and anti-TNF-α (1:400) overnight at 4 °C. The next day, sections were incubated with a biotinylated goat anti-rabbit secondary antibody (1:200) for 50 min at room temperature. The specimens were then examined and photographed under a high-quality microscope. The number and area of positive cells in each sample were counted by Imaging Pro Plus software.

#### ELISA

The animal serums were collected to determine the expression of Acp-5b and OCN via ELISA kits following the manufacturers’ instructions.

#### OC differentiation

Primary bone marrow cells were isolated from the long bones of 8-week-old KO and control mice. Cells were isolated from the femur and tibiae bone marrow and cultured with complete α-MEM in the presence of 10% FBS, 1% penicillin/streptomycin, and 25 ng/ml M-CSF for 24 h. Non-adherent cells were harvested and cultured with fresh medium containing 25 ng/ml M-CSF and 50 ng/ml RANKL (ρ = 1.6 × 10^5^ cells/cm^2^). Cell culture media were replaced every 2 days until mature OCs had formed.

#### TRAP staining and TRAP activity assay

Differentiated cells were stained for TRAP via the TRAP staining kits following the manufacturers’ instructions. TRAP-positive multinucleated cells with at least three nuclei were examined under a microscope and were classified as mature OCs. The supernatant of the OC culture was collected to determine the TRAP activity via the TRAP activity kits following the manufacturers’ instructions.

#### Quantitative real-time PCR

qt-PCR was performed as described^23^. Total RNA was isolated and reverse transcribed to cDNA with M-MuLV reverse transcriptase (TaKaRa). qt-PCR was carried out with AceQ qPCR SYBR Green Master Mix (Vazyme) on the Bio-Rad CFX96 Real-Time PCR Detection System (Bio-Rad) with specific primers. The primer sequences were as follows: QKI (Accession number: NM_001159517.1), 5′-TAGCAGAGTACGGAAAGACATG-3′ (forward) and 5′-GGGTATTCTTTTACAGGCACAT-3′ (reverse); ACP5 (accession number: NM_ 011242384.2), 5′-CCTCTGCAACTCTGGACTCTG-3′ (forward) and 5′-AATCCATCTTGGCGGTGGG-3′ (reverse); CTSK (accession number: NM_ 007802.4), 5′-TCCGAAAAGAGCCTAGCGAA-3′ (forward) and 5′-CCGAGAGATTTCATCCACCTTG-3′ (reverse); NFATc1 (accession number: NM_ 001164111.1), 5′-GCAGAGATTGGAGGCCTTGTG-3′ (forward) and 5′-TAACTGTAGTGTTCTGCGGC-3′ (reverse); c-FOS (accession number: NM_ 021201871.1), 5′-CCCGGCTTTCCCCAAACTT-3′ (forward) and 5′-GCGCAAAAGTCCTGTGTGTT-3′ (reverse); and TRAF6 (accession number: NM_ 009424.3), 5′-GTACGATCGGGTTGTGTGTG -3′ (forward) and 5′-CAAGTTTCCGTGCCAGCATC-3′ (reverse).

#### Western blot analysis

Cells were lysed and centrifuged at 12,000 rpm for 15 min. After quantification of the protein concentration using a BCA kit (Pierce), the same amounts of proteins were resolved on SDS polyacrylamide gels and transferred onto a PVDF membrane (Millipore). The membrane was blocked in 8% nonfat milk with TBST with 0.1% Tween for 2 h at room temperature, and then incubations were performed with the indicated primary antibodies for QKI, NFATc1, c-FOS, CN, NF-κB p65, phospho-NF-κB p65, IκBα, phosphor-IκBα, p38, phospho-p38, JNK, phospho-JNK, ERK, phospho-ERK, and β-actin overnight at 4 °C. After washing with TBST three times for 10 min each, the membrane was incubated with HRP-conjugated secondary antibodies for 1 h at room temperature. Next, the membrane was washed in TBST and detected by the MiniChemi Chemiluminescence imaging system (Sage Creation, Beijing, China).

#### OB differentiation with or without OC CM

Primary bone marrow cells were isolated from the long bones of KO and control mice of 8-week-old in the previous way. Adherent cells were harvested and cultured with fresh medium containing 50 µg/ml ascorbic acid, 10 nM dexamethasone and 10 mM β-glycerophosphate. For the CM, during OC induction, OC supernatant was collected and added into MSC with OB medium (1:1 ratio) to induce OB formation. cell culture media were replaced every 5 days until mature OBs had formed after about three weeks.

#### Alizarin red staining

Differentiated cells were stained for Alizarin red via the Alizarin red staining kits following the manufacturers’ instructions.

#### OVX mouse model

Three groups were formed out of 12‐week‐old female mice: five KO mice treated with a mock operation were the sham group, five control mice with OVX were the control group, and five KO mice with OVX were the OVX group. The KO mice were randomly assigned to sham or OVX group using a random number table. All mice were provided with postoperative recovery 3–5 days and then were given a normal diet and water. After 8 weeks, the lower limb bones and serum were removed. The femurs were fixed in 4% paraformaldehyde and subjected to μCT analysis and histological analysis of OCN immunohistochemical staining and TRAP staining as previously described. Serum were collected to determine the expression of Acp-5b and OCN as previously described.

### Statistical analysis

The investigators who collected and analyzed the data were blinded to the group allocation. All the data obtained were analyzed by SPSS 11.0 software. Investigators used the software program to test the normal distribution and homogeneity of variance of the collected data before further analysis. All the data obtained were presented as the mean ± S.D. using Student’s *t*-test (two comparisons, or two-tailed) and one-way ANOVA (multiple comparisons). *P*-values of <0.05 were considered significant.

## Results

### Loss of OC precursors QKI resulted in decreased bone mass in vivo

**Fig. 1:**
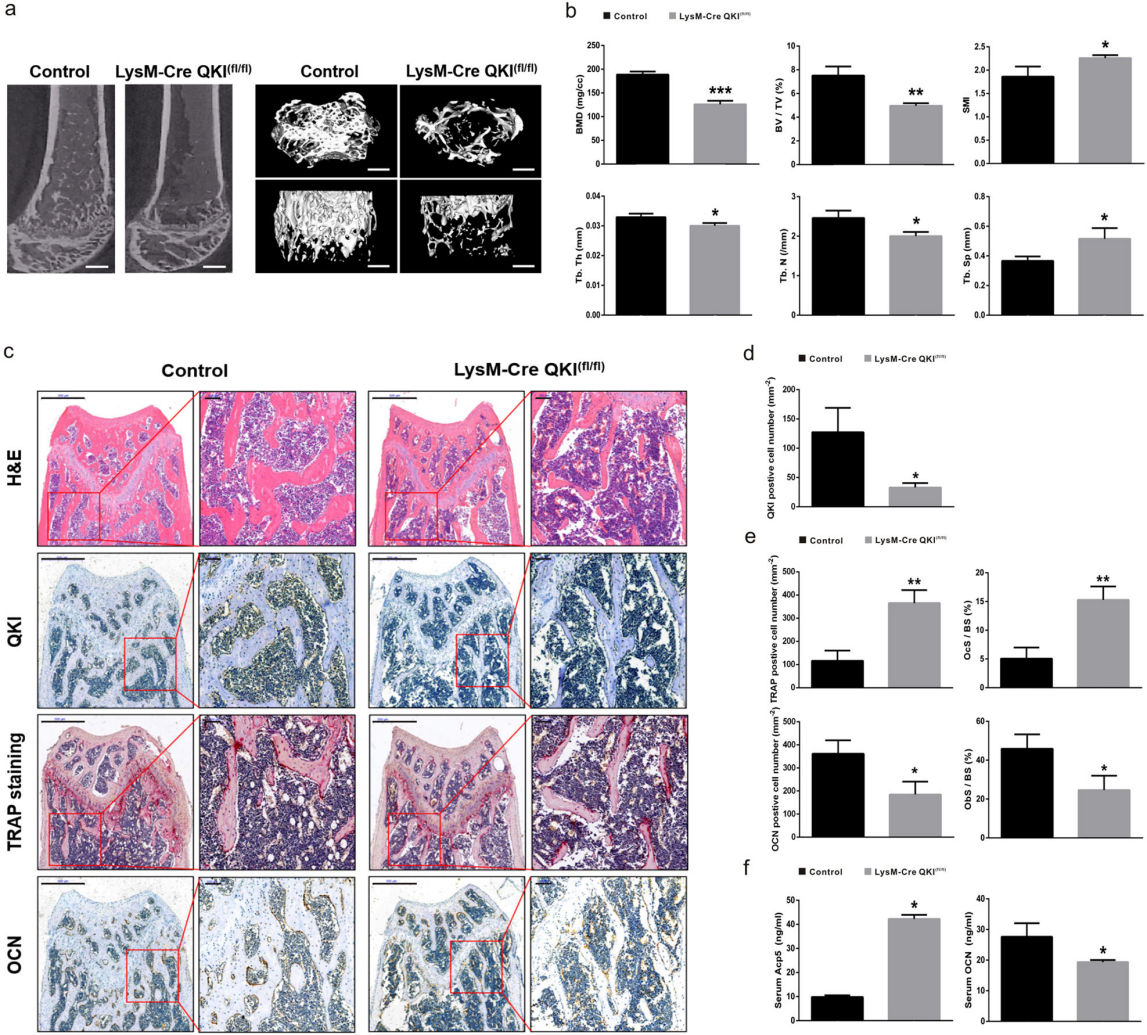
Decreased bone mass and increased OC number in 8-week-old KO mice. **a** Representative μCT reconstructed images of trabecular bone from KO or control group. Scale bar, 200 μm (left image, sagittal view of metaphyseal region; top in right image, horizontal view; bottom, coronal view). **b** Quantification of BMD, BV/TV, Tb.Th, Tb.N, Tb.Sp, and SMI were measured. *n* = 6, *P < 0.05, **P < 0.01, ***P < 0.001. **c** Histological analysis of H&E, TRAP staining, and QKI, OCN IHC in femur metaphyseal region. Scale bar, 500 μm in left image in each group and 100 μm in right image in each group. **d**, **e** The data shown as the mean ± sd. OcS/BS osteoclast surface per bone surface, ObS/BS osteoblast surface per bone surface. *n* = 6, *P < 0.05, **P < 0.01. **f** Serum Acp5 and OCN abundance in KO and control mice. *n* = 6, *P < 0.05.

To determine whether QKI affects the bone mass phenotype especially osteoclastogenesis in vivo, we use our homozygous monocyte/macrophage-specific QKI knockout (LysM-Cre QKI^fl/fl^, KO) transgenic mouse model as experimental mouse^23^. Eight weeks after birth, KO mice showed normal appearance and growth except larger spleen (Supplementary Fig. S1). Then, we carefully examined the bone phenotype of this mouse model by performing micro-computered tomography (μCT) of KO mice and LysM-Cre mice (control mice). Representative bone microstructure imaging (Fig. 1a) of the femoral metaphysis shows significantly decreased cancellous bone mass in KO mice, characterized by reduced bone indices, including bone mineral density (BMD), trabecular bone volume per tissue volume (BV/TV, BVF), trabecular thickness (Tb.Th), and trabecular number (Tb.N), and a concomitant increase in trabecular spacing (Tb.Sp) and structural model index (SMI) (Fig. 1b). Histomorphometric analysis revealed a significantly increased number of TRAP-positive cells and a decreased number of osteocalcin (OCN)-positive cells in KO mice. Together with the bone tissue surface, we considered that compared with control group, there were more OCs and less OBs in KO mice (Fig. 1c–e). In addition, the amount of secreted Acp5 increased, whereas the amount of secreted OCN decreased in the serum of KO mice compared with those of control mice (Fig. 1f). In addition, these differences were also observed in 36-week-old mice (Supplementary Fig. S2). These results suggested that in physiological conditions, KO mice of different ages had an osteopenic phenotype with decreased cancellous bone volume caused by increased OCs and decreased OBs.

### QKI expression decreased during RANKL-stimulated osteoclastogenesis

**Fig. 2:**
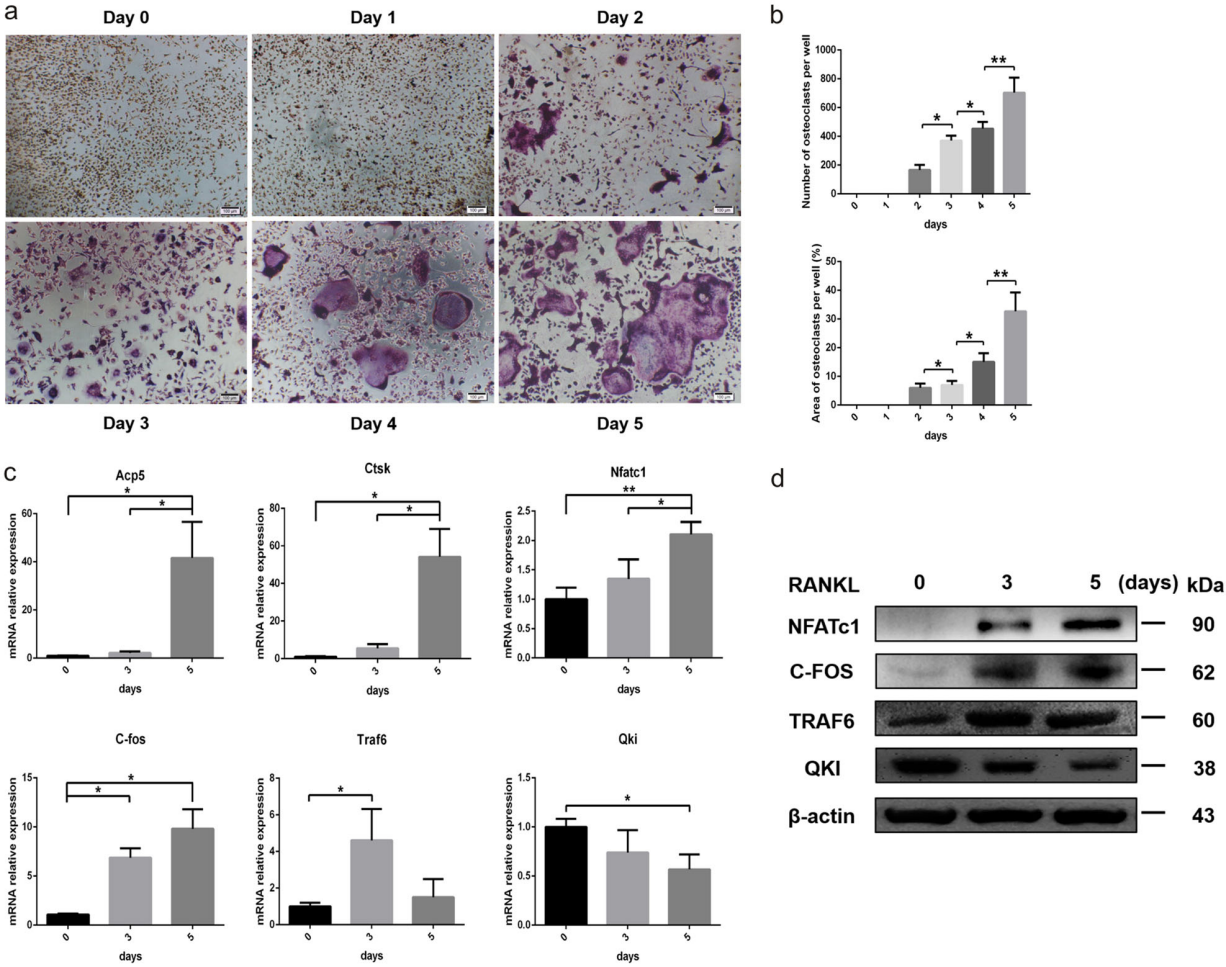
During osteoclastogenesis, QKI expression decreased gradually. **a** TRAP staining was performed in BMMs treated with 25 ng/ml M-CSF and 50 ng/ml RANKL on day 5. Scale bar, 100 μm. **b** The number and area of TRAP-positive cells were counted. *n* = 5, *P < 0.05, **P < 0.01. **c** qt-PCR analysis of OC formation specific genes and Qki gene. *n* = 4, *P < 0.05, **P < 0.01. **d** Western blot analysis of OC formation important transcription factors and QKI expression.

To determine the relationship between QKI expression and OC formation, we used primary bone marrow cells from 8-week-old LysM-Cre mice to induce OCs and then observed changes in QKI expression during the process. TRAP staining results demonstrated that under the stimulation of M-CSF and RANKL, monocytes/macrophages gradually fused and differentiated into OCs (Fig. 2a, b). Quantitative real-time PCR (qt-PCR) analysis showed that the expression levels of readout genes for OC differentiation, including Acp5, Ctsk, Nfatc1, c-fos, and tumor necrosis factor recepto-associated factor 6 (Traf6), were constantly increasing, whereas Qki expression level gradually decreased (Fig. 2c). Moreover, the expression trend of QKI in western blot analysis was also contrary to the expression of these important transcription factors during OC differentiation, such as NFATc1, c-FOS, and TRAF6, further confirming the previous results we obtained at the RNA level (Fig. 2d). Taken together, these data demonstrated that QKI expression was negatively correlated with OC differentiation.

### QKI deficiency stimulated osteoclastogenesis in vitro

**Fig. 3:**
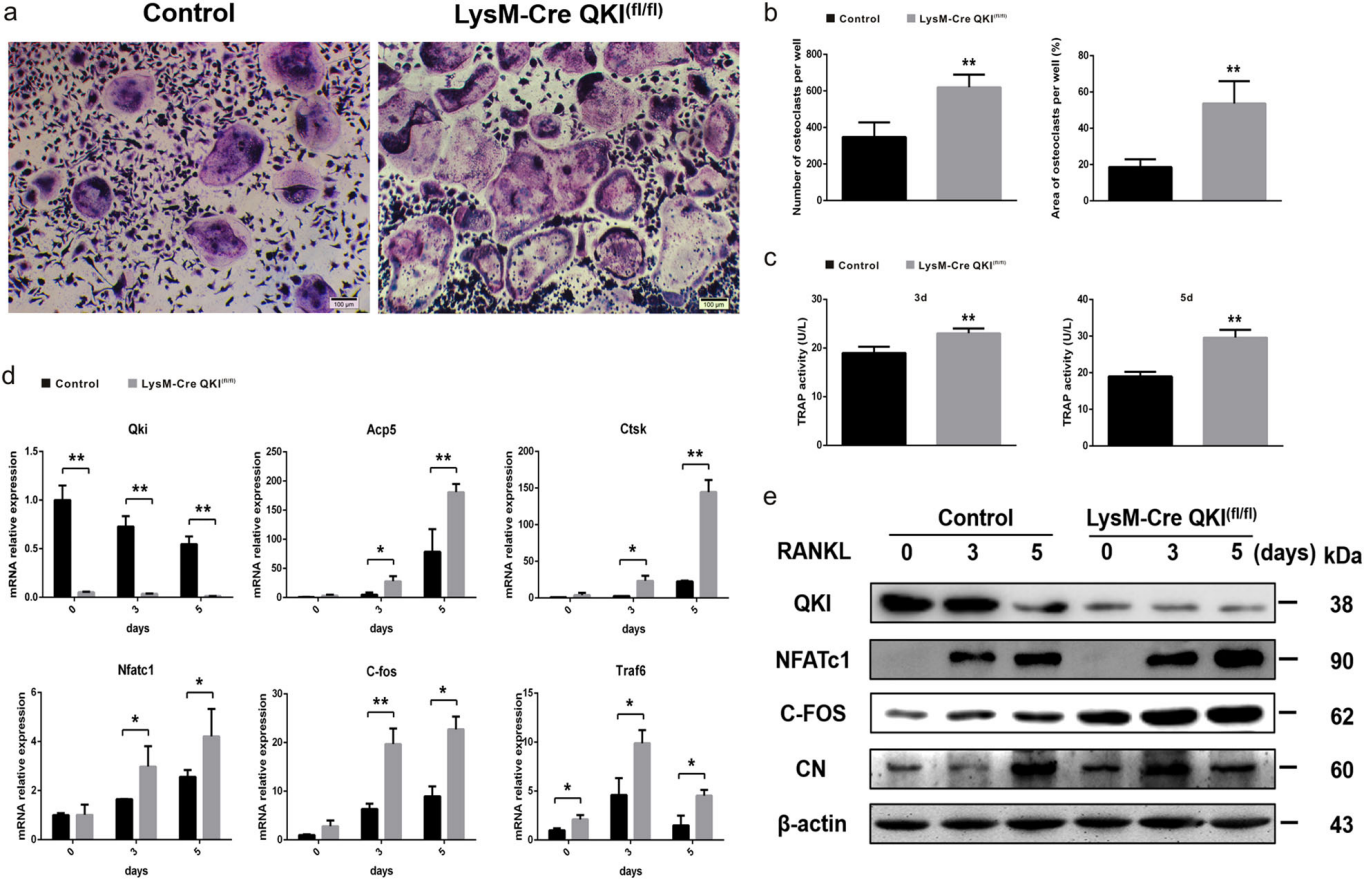
QKI deficiency stimulate osteoclastogenesis. **a** TRAP staining was performed in OCs induced by BMMs from KO or control group treated with 25 ng/ml M-CSF and 50 ng/ml RANKL. Scale bar, 100 μm. **b** The number and area of TRAP-positive cells was counted. *n* = 5, **P < 0.01. **c** TRAP activity assessment using cell supernatant was accomplished on the third and fifth day after BMMs from KO or control group treated with 25 ng/ml M-CSF and 50 ng/ml RANKL. *n* = 4, **P < 0.01. **d** qt-PCR analysis of Qki gene and OCs formation specific genes and important transcription factors expression. *n* = 4, *P < 0.05, **P < 0.01. **e** Western blot analysis of QKI and OC formation important transcription factors between KO and control group on certain day during osteoclastogenesis stimulated with M-CSF and RANKL.

We next examined the effects of QKI deficiency on RANKL-induced OC differentiation in vitro in the presence of M-CSF. OC precursors from bone marrow originating from KO and control mice were cultured with M-CSF and RANKL for 5 days and stained for TRAP. The number of TRAP-positive multinucleated OCs and their areas per well were quantified (Fig. 3a). In stark contrast, we found significantly increased OC numbers as well as OC surface areas (Fig. 3b) in KO mice compared with those in control mice. Consistent with the results of TRAP staining, QKI deficiency also enhanced TRAP activity of OCs both 3 and 5 days after RANKL stimulation (Fig. 3c). In the process of OC formation with M-CSF and RANKL treatment, RNA and protein samples were extracted on 0, 3, and 5 days after RANKL induction, respectively. qt-PCR analysis showed that knockout of QKI led to the expression levels of these OC-related genes markedly increased in KO group compared with control group (Fig. 3d). Correspondingly, the expression levels of NFATc1, C-FOS, and CN, the key regulators of OC differentiation, were increased during RANKL-treated OC formation in QKI-deficient bone marrow macrophages (BMMs) (Fig. 3e). Therefore, these results showed that OC differentiation increased when QKI level was low, suggesting that QKI has a critical role in osteoclastogenesis.

### Knockout of QKI activates the NF-κB and MAPK signaling pathways during osteoclastogenesis

**Fig. 4:**
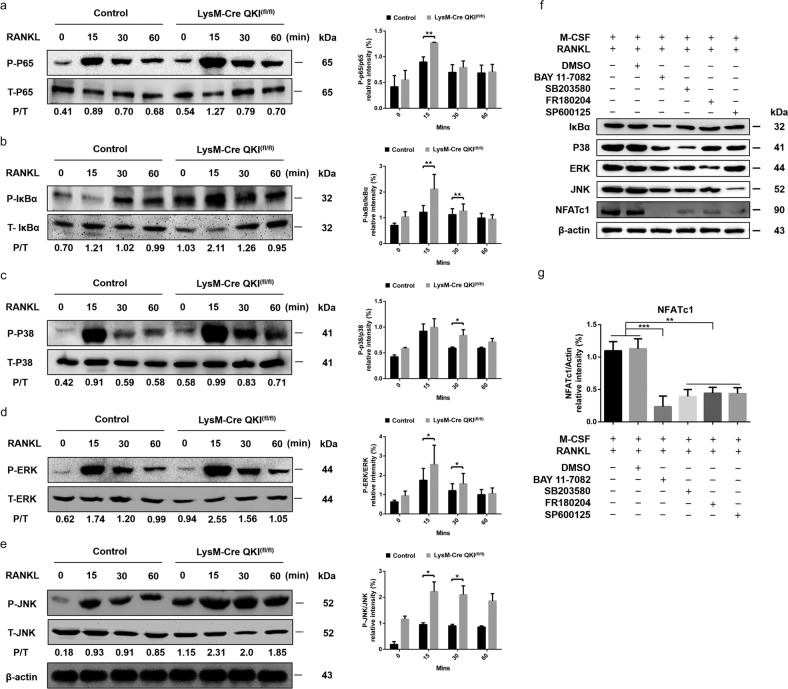
QKI deficiency amplified the activation of the NF-κB and MAPK signaling pathways during osteoclastogenesis. **a**–**e** Western blot analysis of RANKL-induced p65, IκBα, p38, ERK, JNK in BMMs from KO and control mice, and quantitative analysis of the western blot data, respectively. *n* = 4, *P < 0.05, **P < 0.01. **f** Western blot analysis of NFATc1 expression after blocking IκBα, p38, ERK, and JNK by inhibitors. **g** Quantitative analysis of the western blot data evaluating NFATc1 expression after blocking signaling pathways. *n* = 4, **P < 0.01, ***P < 0.001.

To clarify the precise mechanisms of osteoclastogenesis upregulation by QKI deficiency, western blot analysis was used to monitor the major RANKL-dependent endogenous signaling pathways involved in OC formation. Protein samples were extracted in various stages (0, 15, 30, and 60 min). Interestingly, the western blot analysis results showed that after exposure to RANKL, the activation of NF-κB P65, and IκBα was enhanced substantially within 60 min in KO group cells. In addition, knockout of QKI also led to a slight upregulation of phosphorylation of the three typical MAP kinases (P38, ERK, and JNK) (Fig. 4a–e). In order to determine that the acceleration of QKI deficiency on osteoclastogenesis was through the effect on NF-κB and MAPKs pathways, we used various antagonists (NF-κB inhibitor BAY11-7082, p38 inhibitor SB203580, JNK inhibitor SP600125 and ERK inhibitor FR180204) to test the effects of blocking these signaling pathways on the expression of NFATc1, the role transcription factor in osteoclastogenesis. The results showed that under the stimulus of M-CSF and RANKL, blocking these signaling pathways regulating osteoclast formation above, NFATc1 expression distinctly weakened. Furthermore, we found that inhibitor BAY11-7082 predominate inhibition effect was most obvious (Fig. 4f, g). These results indicated that the NF-κB and MAPKs signaling pathways were involved in the effects on osteoclastogenesis by QKI deficiency. After blocking these pathways, the original promoting effects had been significantly lessened. Thus, these results at the protein level suggested that QKI deficiency might facilitate OC formation by affecting and amplifying the signal cascades on the NF-κB and MAPK pathways, which had vital roles in osteoclastogenesis.

### QKI deficiency aggravated the imbalance in to OB-OC crosstalk in bone metabolism

**Fig. 5:**
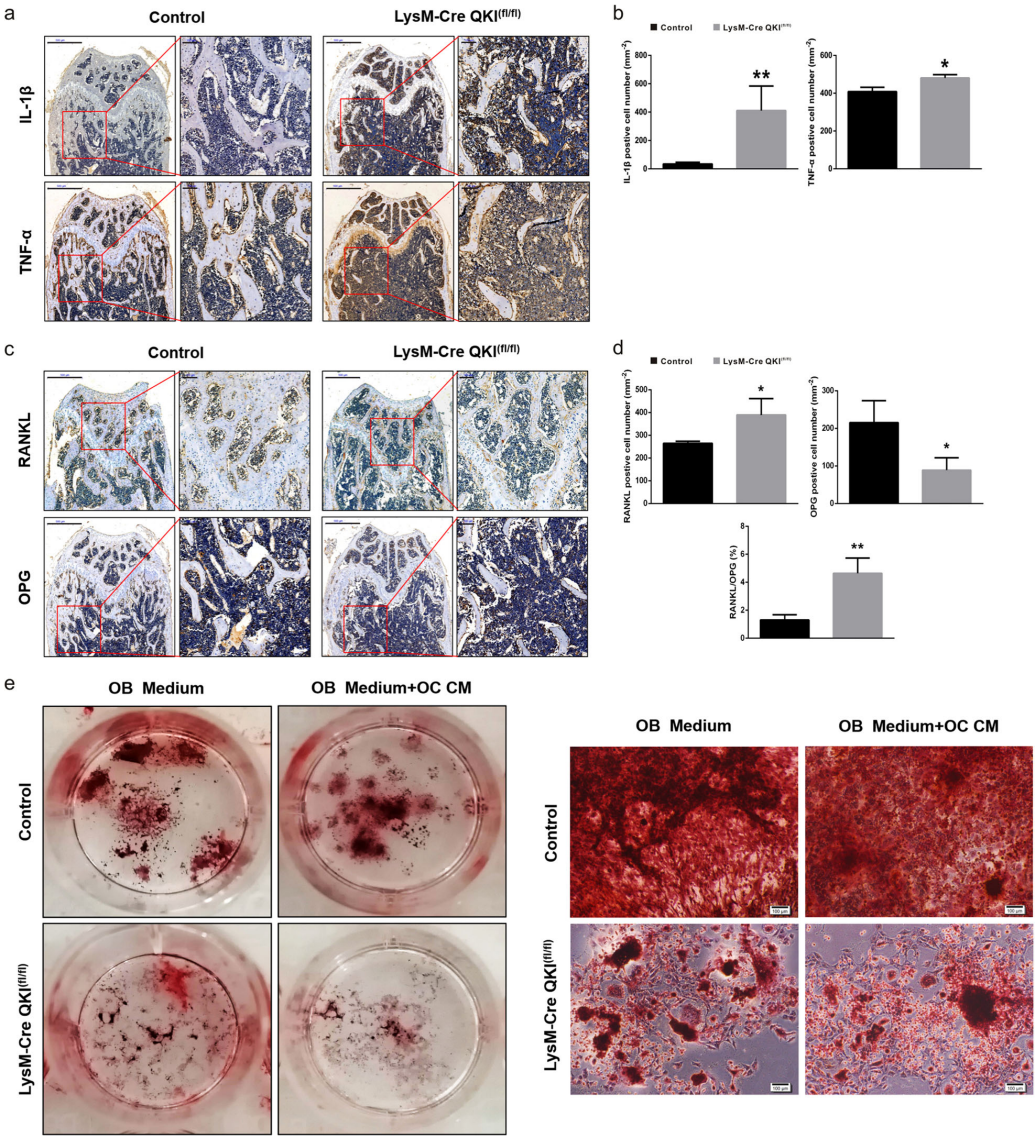
QKI deficiency aggravated the imbalance in OB-OC crosstalk in bone metabolism in 8-week-old KO mice. **a** Histological analysis of IL-1β and TNF-α IHC in femur metaphyseal region. Scale bar, 500 μm in left and 100 μm in right. **b** The data shown as the mean ± sd. *n* = 6, *P < 0.05, **P < 0.01. **c** Histological analysis of RANKL and OPG IHC in femur metaphyseal region. Scale bar, 500 μm in left and 100 μm in right. **d** The data shown as the mean ± sd. *n* = 6, *P < 0.05, **P < 0.01. **e** Induced OBs were stained by Alizarin red staining. Scale bar, 100 μm.

We previously found an imbalance between OCs and OBs in the bone metabolism of KO mice. Considering the coupling effects of OCs and OBs on bone homeostasis, we suspected that although we only knocked out QKI from the monocyte/macrophage lineage, there was something suppressing the growth of OBs and breaking the equilibrium. Investigations were made to explore the effects of QKI deficiency on the OB-OC crosstalk in bone metabolism. Immunohistochemical staining of interleukin-1β (IL-1β) and tumor necrosis factor (TNF)-α was used to evaluate the femoral medullary inflammation between KO and control mice. The results showed that there were much more IL-1β- and TNF-α-positive cells in KO group, which indicated that the intraosseous inflammation of KO mice was more severe (Fig. 5a, b). As inflammatory environments inhibited OB formation, this might be one of the reasons for the decrease in the number of OBs in KO group. In addition to the factor that QKI deficiency could promote OC differentiation to increase the number of OCs, we also learned from the immunohistochemical results of RANKL and osteoprotegerin (OPG) that in KO mice, the number of RANKL-positive cells increased and the number of OPG-positive cells decreased, suggesting that the increased OCs in KO group were also related to the increased ratios of RANKL and OPG (Fig. 5c, d). These finding were also observed in 36-week-old mice (Supplementary Fig. S3). In addition, to assess the possible coupling interactions between OCs and OBs to the bone formation response in vitro, OC culture derived from KO or control mice were used as conditioned medium (CM) and added into the OB induction using primary bone marrow cells from each group. We found that in the presence of CM, the number of OBs induced in KO group was significantly lower than that in control group (Fig. 5e). Taken together, these data demonstrated that in addition to directly promoting OC differentiation, QKI deficiency could promote OC formation by increasing the RANKL/OPG ratio and could also inhibit OB formation by impacting the local inflammatory microenvironment. In addition, differentiated OCs of KO mice might secrete some factors to inhibit OB production.

### Loss of QKI led to more serious osteopenia under pathological status

**Fig. 6:**
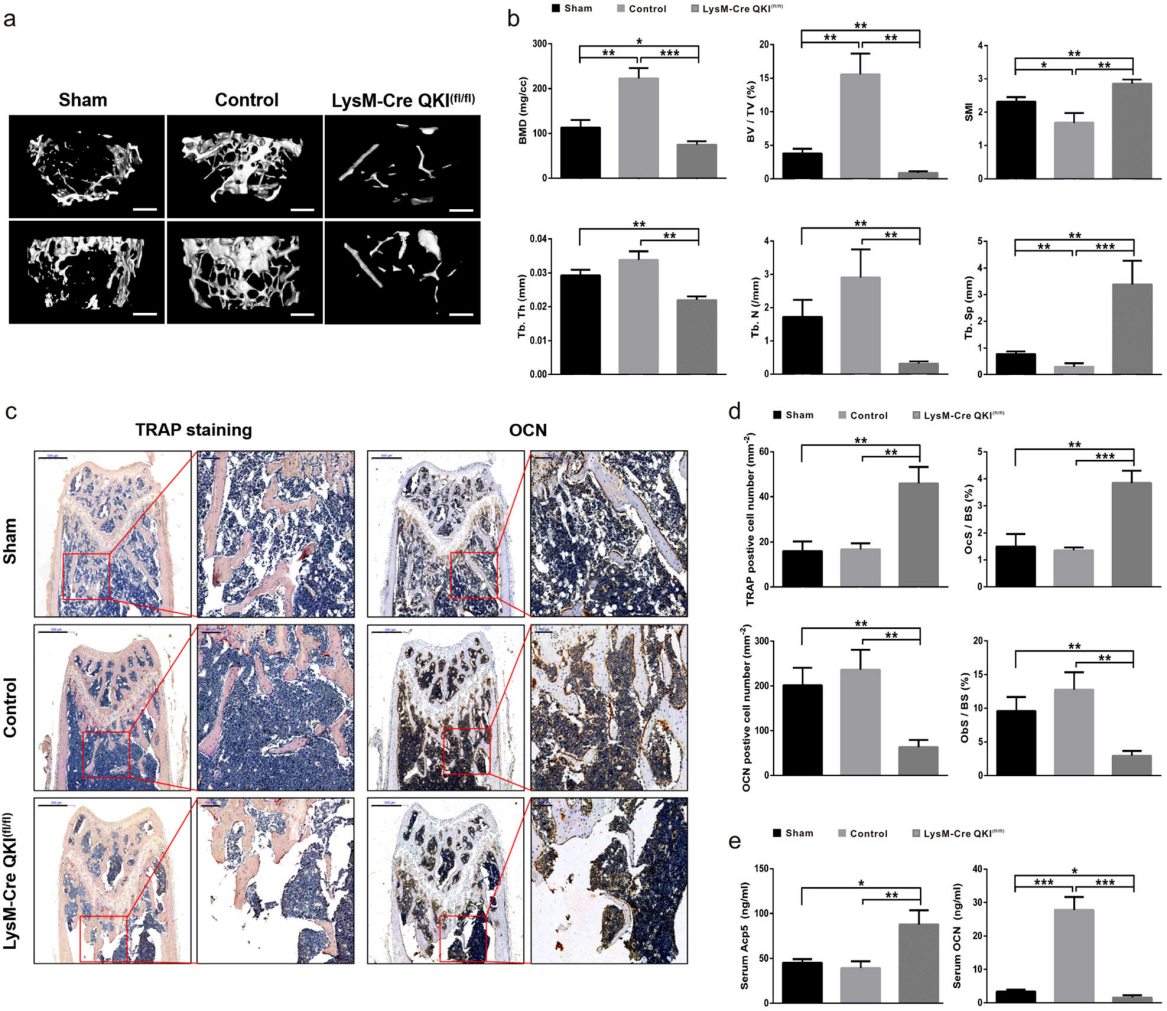
QKI deficiency led to more serious osteopenia in OVX mice model. **a** Representative μCT reconstructed images of trabecular bone from sham, control and KO group. Scale bar, 200 μm (top, horizontal view; bottom, coronal view). **b** Quantification of BMD, BV/TV, Tb.Th, Tb.N, Tb.Sp, and SMI were measured. *n* = 5, *P < 0.05, **P < 0.01, ***P < 0.001. **c** Histological analysis of TRAP staining and OCN IHC in femur metaphyseal region. Scale bar, 500 μm in left and 100 μm in right. **d** The data shown as the mean ± sd. *n* = 5, **P < 0.01, ***P < 0.001. **e** Serum Acp5 and OCN abundance in sham, control, and KO group. *n* = 5, *P < 0.05, **P < 0.01, ***P < 0.001.

In previous experiments, we found that QKI-deficient mice had a phenotype of decreased bone mass under physiological conditions. Then, to investigate the effects of QKI deficiency on the bone mass change under pathological conditions, such as osteoporosis after losing estrogen protection, an ovariectomy (OVX) mouse model was established (Supplementary Fig. S4). Twelve-week-old KO and control mice were ovariectomized, and an extra KO mice group with same age received a mock operation. Eight weeks later, the femurs were collected for μCT scanning. The analysis results showed that KO OVX mice had more serious osteopenia than KO sham mice and control OVX mice (Fig. 6a). Quantitative analysis showed that although the SMI and Tb.Sp were markedly increased, the BMD, BV/TV, Tb.Th, and Tb.N were significantly decreased in KO OVX mice compared with the other groups (Fig. 6b). To further confirm the changes of KO mice with OVX treatment in osteoclastogenesis and osteoblastogenesis in vivo, we also performed histological OCN and TRAP staining. The results showing that increased number of TRAP-positive cells and decreased number of OCN-positive cells obviously indicated that there were more OCs and fewer OBs in KO OVX mice than the other groups (Fig. 6c, d). In addition, the amount of secreted Acp5 increased, whereas the amount of secreted OCN decreased in the serum of KO OVX mice compared with the other groups (Fig. 6e). Therefore, these data demonstrated that QKI deficiency aggravated OVX-induced bone loss, causing more serious osteoporosis.

## Discussion

QKI is a member of the STAR family. The functions of QKI have been proved to include promoting oligodendroglia differentiation^12,13^, stimulating endothelial cell and smooth muscle cell proliferation and differentiation^11^, inhibiting intestinal epithelial cell proliferation but promoting its differentiation^14^, inducing monocyte differentiation^22^, and controlling myofibril formation^18^. In addition, a recent study using a homozygous myeloid lineage-specific QKI knockout transgenic mouse model indicated a novel role for QKI in restraining the LPS-mediated inflammatory response through the biphasic regulation of macrophage polarization^23^.

In this study, we first used the mouse model abovementioned to observe changes in bone mass. The model exhibits a cancellous osteopenic phenotype in distal femur metaphysis, similar to what is observed in some forms of osteoporosis. Quantitative analysis results of μCT showed that the values of KO mice trabecular BMD and BVF were significantly lower than those of control mice; these decreased values were related to the thinner and fewer trabecula of KO mice. In addition, the appearance of cancellous osteopenia was not only observed in 8-week-old KO mice but also in 36-week-old mice. In Ferguson’s study, the bone mass of C57/BL6 mice showed continuously increased until 42 weeks after birth^24^. The continuous increase in bone mass in control group and the continuous inhibition of bone formation in KO group made the difference between the two groups of 36-week-old mice more pronounced. Unlike Kim’s findings^25^, in our study, both male and female mice showed the same change in the trabecular bone, demonstrating that the impact of the QKI deficiency has a non-sex-specific response. The amount of bone mass depends on the activity of OCs and OBs, so the reduced bone mass in KO mice hinted at an imbalance between the two important cells in bone metabolism. As TRAP and OCN were considered to be OCs and OBs biological markers, respectively^26^, to understand the cause of osteoporosis, TRAP and OCN staining were used to identify OCs and OBs. Bone histology staining showed that the decrease in cancellous bone mass in KO mice was caused by the increase in OCs and the decrease in OBs in bone tissue. ELISA analysis results of Acp-5b and OCN in mouse peripheral blood serum further confirmed this conclusion.

Wang’s study^23^ indicated that QKI deficiency affected macrophage polarization. Together with the origin of OCs and our results above, we speculated there is a relationship between the absence of QKI and OC formation. However, little known about the effects and mechanism of QKI in osteoclastogenesis. Therefore, based on these considerations, we believe that it is particularly important to study the effects of QKI deficiency on OCs.

Through TRAP staining and the detection of OC-specific markers, we determined that we could effectively induce monocyte macrophages to differentiate into OCs by co-stimulation with M-CSF and RANKL. From the third day after RANKL treatment, the OC number and area started to increase and increased significantly on the fifth day, which was consistent with Kim’s report on OC induction^3^. Besides of ACP5, CTSK is regarded as a good marker reflecting OC activity^27^. NFATc1, TRAF6, and C-FOS are important transcription factors in the process of OC proliferation, differentiation and function^28,29^. The expression levels of the abovementioned markers could reflect the degree of OC differentiation and function. These markers play the different roles in different stages of OC generation, so the time nodes at which the marker expression peak might be different^5^. However, in general, as reported by others, we found that the expression level of these markers increased with OC progressive generation. In addition, we also detected the expression level of QKI during OC formation process. Mirroring the high expression levels of these OC markers, the expression of QKI decreased only with osteoclastogenesis, which was contrary to the trend of OC formation. These results were also verified at protein level detection.

With the in vivo phenotype and the relationship between QKI and OCs, we needed to verify the role of QKI in vitro. Consistent with the results above, we found that more primary bone marrow cells differentiated into OCs in KO mice than in control mice after the same RANKL stimulation, even the mature OC surface areas were much larger. The expression levels of marker genes were markedly upregulated in KO mice. In terms of protein level, both the expression levels of transcription factors and the activity degree of TRAP indicated that the absence of QKI had an obvious role in promoting the formation of OCs.

In the process of OC formation, RANKL binding to its receptor RANK leads to the activation of downstream signaling molecules, such as NF-κB and MAPK components (P38, JNK, and ERK1/2), and NFATc1 is the convergence point of the multiple pathways^6,8,28–32^. To determine the molecular mechanism by which QKI drives the regulation of OC differentiation, we incubated primary bone marrow cells from KO and control mice in the presence of M-CSF and RANKL, and monitored the activation of endogenous signaling components following treatment. We found that compared with control group, KO group cells had higher levels of phosphorylated p65, IκBα, p38, JNK, and ERK within 60 min after RANKL-treated, indicating that QKI deficiency might promote OC differentiation by triggering and amplifying intermolecular cascades in the pathways to varying degrees. In addition, subsequent experiments with inhibitors further confirmed these data previous. These results are consistent with the inhibitory effects of QKI on NF-κB and c-fos during inflammation and the cell cycle^20,23^.

Moreover, OBs are involved in bone-building function and are linked to the maintenance of bone homeostasis^33^. However, in our study, OB formation was suppressed, which hinted an uncoupling of OBs and OCs in KO mice. Considering that we only knocked out QKI in myeloid lineage cells, QKI deficiency could not directly affect OBs. Thus, the possible influence of gene absence on OBs was investigated. We found that after QKI knocked out from the myeloid lineage, there were a large amount of inflammatory factor infiltration in the bone marrow cavity. This is consistent with our previous results about the effects of QKI on inflammation^23^. Inflammatory cytokines, such as TNF, could cause a bone turnover imbalance of OC induction and OB inhibition^34^. Therefore, in such an inflammatory microenvironment, OB formation was inhibited. In addition, we found that in the presence of OC CM, OB formation in KO mice was significantly suppressed. HAN revealed that osteocytes, OBs and OCs interact with each other through endocrine and paracrine action^35^. Thus, we supposed that the OCs in KO mice might secrete factors that inhibit OBs, and the negative effects of these factors on OBs exceed the induction of osteogenesis.

The RANK/RANKL/OPG system is widely recognized as an important signal transduction pathway for maintaining the OC differentiation and bone metabolism balance^36,37^. The RANKL/OPG ratio is related to the differentiation of OCs. As the ratio increases, OC production increases, and vice versa^38,39^. According to the immunohistochemical results of RANKL and OPG, the ratio of RANKL/OPG in KO group was significantly increased. This imbalance might be another microenvironment-related reason to explain pro-osteoclastogenesis in addition to the direct promotion of OC differentiation by QKI deficiency.

We have previously shown that a lack of QKI leads to a decrease in bone mass under physiological conditions. To identify the possible consequences of the loss of QKI in pathological conditions, such as osteoporosis, and to further validate the influence of QKI deficiency on bone mass, we simulated the postmenopausal status of women through OVX, in which the body loses the protective effects of estrogen, bone homeostasis is destroyed, and bone mass is reduced, and then lead to osteoporosis^40^. In our study, we were unable to determine the comparison between sham group and control group because these were two different methods to cause osteopenia. However, we found that KO OVX group had a much more severe reduction in bone mass than control group. This result suggested that the osteopenia caused by QKI deficiency and the effects of pathological conditions on bone mass could be superimposed.

Taken together, our study demonstrated that a QKI deficiency could aggravate osteopenia, and the mechanisms mainly included QKI deficiency increasing the local RANKL/OPG ratio to activate OC differentiation stimulated by RANKL, promoting OC formation by amplifying the signaling cascade of NF-κB and MAPK pathways, and inhibiting OB formation via the inflammatory microenvironment. In addition, we also found that a QKI deficiency would exacerbate pathological osteopenia, suggesting that a QKI deficiency could be considered a possible risk factor for osteoporosis.

## Supplementary information

Supplementary Figure 1

Supplementary Figure 2

Supplementary Figure 3

Supplementary Figure 4

Supplementary Figure Legends
