# Supplementary figures and images for: QKI deficiency leads to osteoporosis by promoting RANKL-induced osteoclastogenesis and disrupting bone metabolism
Source: Cell Death Dis. 2020 May 7;11(5):330. doi: 10.1038/s41419-020-2548-3 (PMC7205892; doi:10.1038/s41419-020-2548-3)

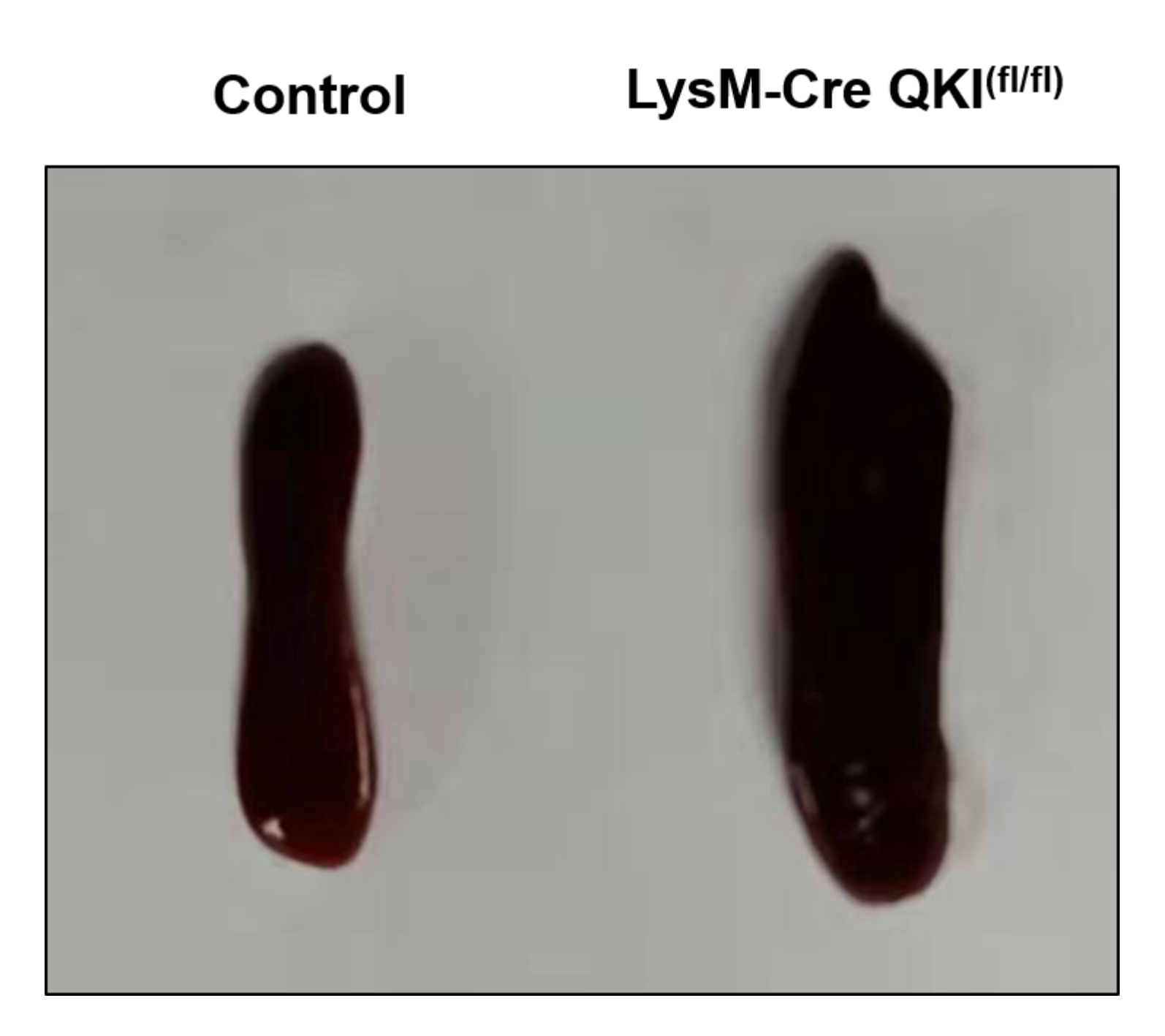

Supplement: Supplementary file 1 — Supplementary Figure 1 [file 41419_2020_2548_MOESM1_ESM.tif]

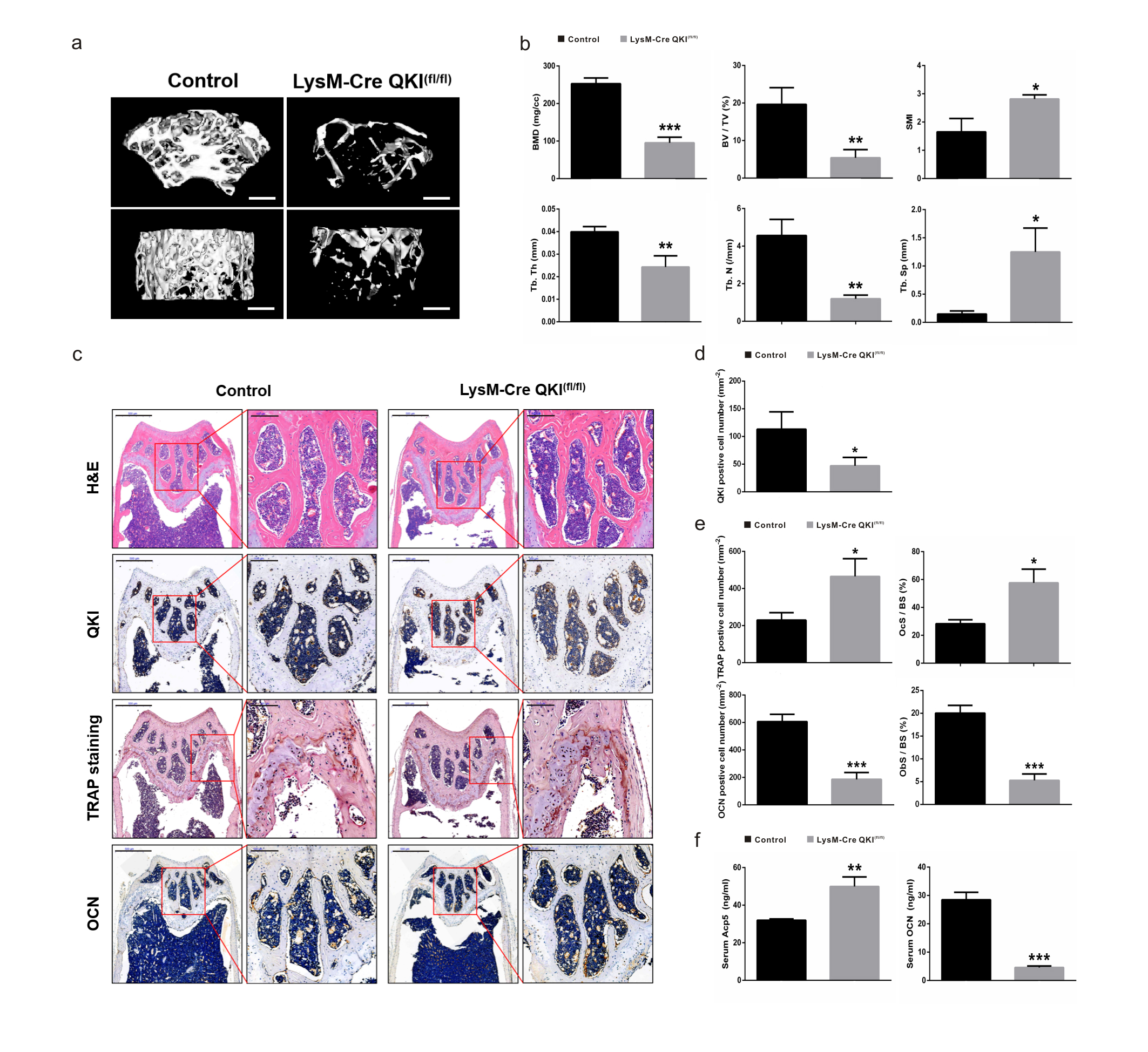

Supplement: Supplementary file 2 — Supplementary Figure 2 [file 41419_2020_2548_MOESM2_ESM.tif]

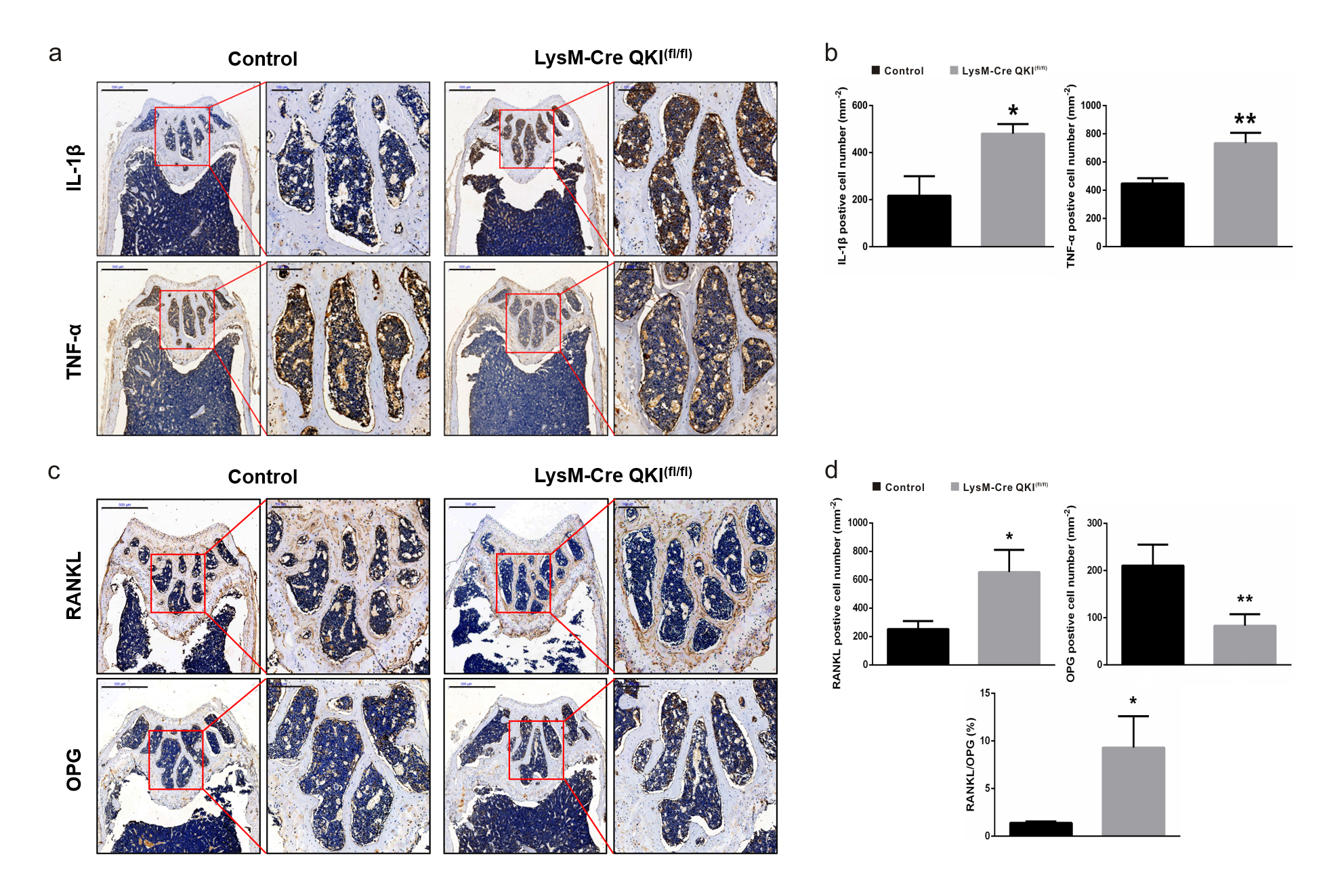

Supplement: Supplementary file 3 — Supplementary Figure 3 [file 41419_2020_2548_MOESM3_ESM.tif]

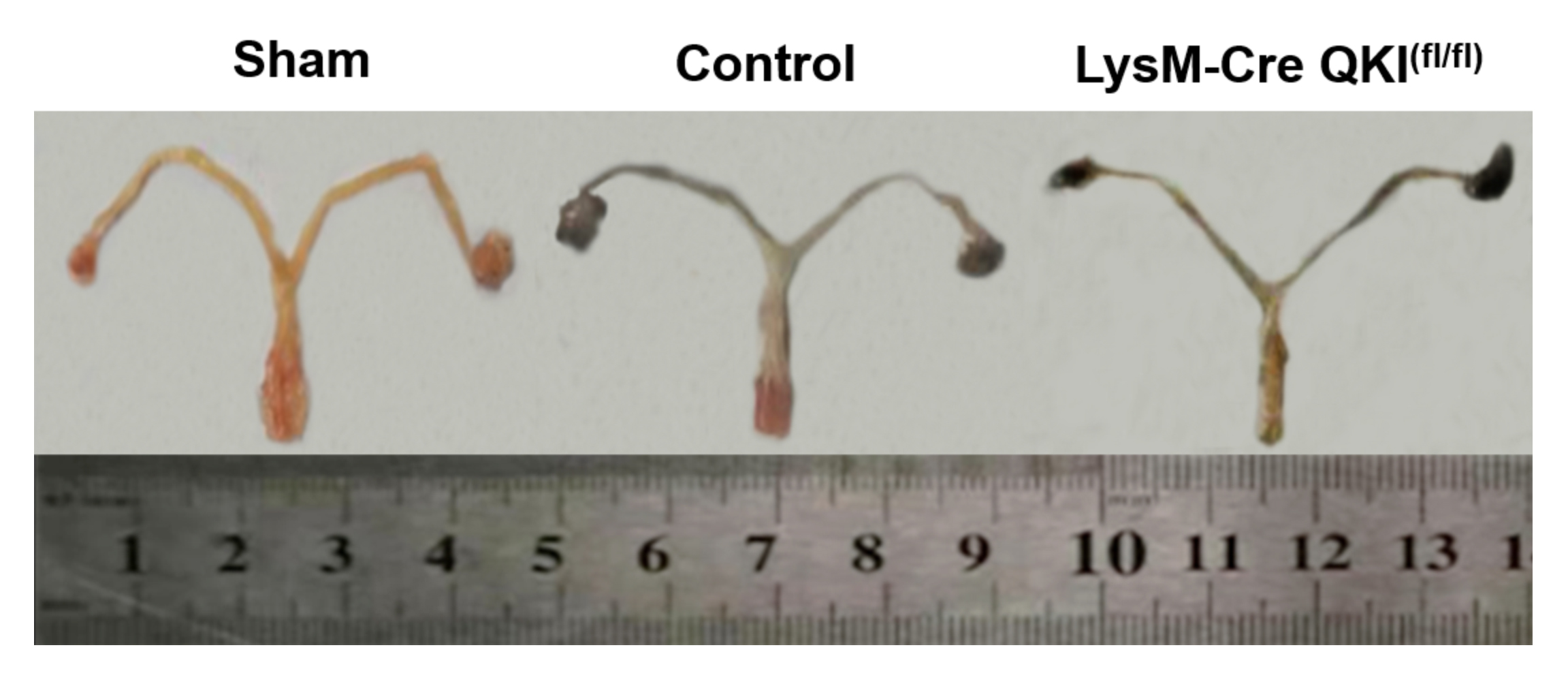

Supplement: Supplementary file 4 — Supplementary Figure 4 [file 41419_2020_2548_MOESM4_ESM.tif]
