# Supplementary material for: QKI deficiency leads to osteoporosis by promoting RANKL-induced osteoclastogenesis and disrupting bone metabolism
Source: Cell Death Dis. 2020 May 7;11(5):330. doi: 10.1038/s41419-020-2548-3 (PMC7205892; doi:10.1038/s41419-020-2548-3)
Supplement: Supplementary file 5 — Supplementary Figure Legends [file 41419_2020_2548_MOESM5_ESM.docx]

**Figure S1**

KO mice had a larger spleen than control mice. n=8.

**Figure S2** Decreased bone mass and increased OC number in 36-week-old KO mice. (a) Representative μCT reconstructed images of trabecular bone from KO or control group. Scale bar, 200μm (top, horizontal view of metaphyseal region; bottom, coronal view). (b) Quantification of BMD, BV/TV, Tb.Th, Tb.N, Tb.Sp and SMI were measured. n=5, *P<0.05, **P<0.01, ***P<0.001.(c) Histological analysis of H&E, TRAP staining and QKI, OCN IHC in femur metaphyseal region. Scale bar, 500μm in left and 100μm in right. (d-e) The data shown as the mean±sd. n=5, *P<0.05, ***P<0.001. (f) Serum Acp5 and OCN abundance in KO and control mice. n=5, **P<0.01, ***P<0.001.

**Figure S3** QKI deficiency aggravated the imbalance in OB-OC crosstalk in 36-week-old KO mice. (a) Histological analysis of IL-1β and TNF-α IHC in femur metaphyseal region. Scale bar, 500μm in left and 100μm in right. (b) The data shown as the mean±sd. n=5, *P<0.05, **P<0.01. (c) Histological analysis of RANKL and OPG IHC in femur metaphyseal region. Scale bar, 500μm in left and 100μm in right. (d) The data shown as the mean±sd. n=5, *P<0.05, **P<0.01.

**Figure S4** Size and morphology of the uterus in each group 8 weeks after the model started to establish. n=6.
